# Supplementary material for: Gender-specific design and effectiveness of non-pharmacological interventions against cognitive decline and dementia–protocol for a systematic review and meta-analysis
Source: PLoS One. 2021 Aug 27;16(8):e0256826. doi: 10.1371/journal.pone.0256826 (PMC8396713; doi:10.1371/journal.pone.0256826)
Supplement: S1 Appendix — (DOCX) [file pone.0256826.s002.docx]

**Gender-specific design and effectiveness of non-pharmacological interventions against cognitive decline and dementia – Protocol for a systematic review and meta-analysis.**

**S1 Appendix: Search strategy**

**PubMed**

#1 prevent*[tiab] OR intervention[tiab] OR regimen[tiab] OR enhanc*[tiab] OR “behavior chang*”[tiab] OR train*[tiab] OR program[tiab] OR modif*[tiab] OR protect*[tiab]

#2 "older age"[tiab] OR older[tiab] OR "middle aged"[tiab] OR "older adult*"[tiab] OR elder*[tiab] OR senior*[tiab]

#3 "dementia"[MeSH] OR "Alzheimer disease"[Majr] OR "Neurocognitive Disorders"[Majr] OR "Neurodegenerative Diseases"[Majr:NoExp] OR "Cognitive Dysfunction"[Majr:NoExp]

#4 dementia[tiab] OR Alzheim*[tiab] OR cognit*[tiab] OR "Cognitive Dysfunction"[tiab] OR memory*[tiab] OR "cognitive aging"[tiab] OR “cognitive decline”[tiab] OR “cognitive impairment”[tiab] OR "memory loss"[tiab] OR "cognitive complain*"[tiab]

**#5 "randomized controlled trial"[pt] OR "controlled clinical trial"[pt] OR "pragmatic clinical trial"[pt] OR "intervention study"[tiab] OR "pilot trial"[tiab] OR "crossover trial"[tiab] OR randomized[tiab] OR randomly[tiab]**

**#3 OR #4 🡪 #6**

**#1 AND #2 AND #6 AND #5**

**PsycINFO**

#1 AB (elder* OR aged OR adult* OR middle-aged OR older adult*) OR TI (elder* OR aged OR adult* OR middle-aged OR older adult*)

#2 DE "Behavior Modification" OR DE "Prevention" OR DE "Intervention"

#3 AB (regimen OR program OR train* OR prevent* OR intervention OR enhanc* OR protect* OR modif*) OR TI (regimen OR program OR train* OR prevent* OR intervention OR enhanc* OR protect* OR modif*))

#4 DE "Alzheimer's Disease" OR DE "Dementia" OR DE "Cognitive Impairment" OR DE "Memory" OR DE "Cognitive Aging" DE "Memory Disorders"

#5 AB (dementia OR alzheimer’s OR alzheimer OR “cognitive impairment” OR “cognitive decline” OR “cognitive dysfunction” OR cognition* OR “memory loss” OR memory) OR TI (dementia OR alzheimer’s OR alzheimer OR “cognitive impairment” OR “cognitive decline” OR “cognitive dysfunction” OR cognition* OR “memory loss” OR memory))

#6 DE "Clinical Trials" OR DE "Randomized Clinical Trials" OR DE "Randomized Controlled Trials"

#7 AB (“randomized controlled trial” OR trial OR rct OR “controlled clinical trial” OR “crossover trial” OR “clinical trial” OR “pilot trial” OR “intervention study” OR "pragmatic clinical trial") OR TI (“randomized controlled trial” OR trial OR rct OR “controlled clinical trial” OR “crossover trial” OR “clinical trial” OR “pilot trial” OR “intervention study” OR "pragmatic clinical trial"))

#2 OR #3 🡪 #8

#4 OR #5 🡪 #9

#6 OR #7 🡪 #10

#1 AND #8 AND #9 AND #10

**Web of Science Core Collection**

#1 AB=(elder* OR "middle-aged" OR older OR senior*) OR TI=(elder* OR "middle-aged" OR older OR senior*)

#2 AB=(“randomized controlled trial” OR trial OR rct OR “controlled clinical trial” OR “crossover trial” OR “clinical trial” OR “pilot trial” OR “intervention study” OR "pragmatic clinical trial") OR TI=(“randomized controlled trial” OR trial OR rct OR “controlled clinical trial” OR “crossover trial” OR “clinical trial” OR “pilot trial” OR “intervention study” OR "pragmatic clinical trial")

#3 AB=(dementia OR alzheimer’s OR alzheimer OR “cognitive impairment” OR “cognitive decline” OR “cognitive dysfunction” OR cognition* OR “memory loss” OR memory) OR TI=(dementia OR alzheimer’s OR alzheimer OR “cognitive impairment” OR “cognitive decline” OR “cognitive dysfunction” OR cognition* OR “memory loss” OR memory)

#4 AB=(regimen OR program OR train* OR prevent* OR intervention OR enhanc* OR protect* OR modif*) OR TI=(regimen OR program OR train* OR prevent* OR intervention OR enhanc* OR protect* OR modif*)

#1 AND #2 AND #3 AND #4

**Cochrane CENTRAL**

ID Search Hits

#1 MeSH descriptor: [Dementia] explode all trees

#2 MeSH descriptor: [Alzheimer Disease] explode all trees

#3 MeSH descriptor: [Neurocognitive Disorders] this term only

#4 MeSH descriptor: [Neurodegenerative Diseases] this term only

#5 (dementia):ti,ab

#6 (Alzheim*):ti,ab

#7 (cognition):ti,ab

#8 ("cognitive dysfunction"):ti,ab

#9 ("cognitive aging"):ti,ab

#10 (memory):ti,ab

#11 ("cognitive decline"):ti,ab

#12 ("cognitive impairment"):ti,ab

#13 ("cognitive complaint*"):ti,ab

#14 ("memory loss"):ti,ab

#15 (older):ti,ab

#16 ("middle aged"):ti,ab

#17 (elder*):ti,ab

#18 (senior*):ti,ab

#19 (prevent*):ti,ab

#20 (intervention):ti,ab

#21 (regimen):ti,ab

#22 (enhanc*):ti,ab

#23 (“behavior chang*”):ti,ab

#24 (train*):ti,ab

#25 (program):ti,ab

#26 (modif*):ti,ab

#27 (protect*):ti,ab

#28 ("randomized controlled trial"):pt

#29 ("controlled clinical trial"):pt

#30 ("pragmatic clinical trial"):pt

#31 ("intervention study"):ti,ab

#32 ("pilot trial"):ti,ab

#33 ("crossover trial"):ti,ab

#34 #28 OR #29 OR #30

#35 #31 OR #32 OR #33

#36 #34 OR #35

#37 {OR #19-#27}

#38 {OR #15-#18}

#39 {OR #5-#14}

#40 {OR #1-#4}

#41 #39 OR #40

#42 {AND #36, #37, #38, #41}

**ALOIS**

Keyword search: *prevent*; study type: intervention study
