# Supplementary material for: Gender-specific design and effectiveness of non-pharmacological interventions against cognitive decline and dementia–protocol for a systematic review and meta-analysis
Source: PLoS One. 2021 Aug 27;16(8):e0256826. doi: 10.1371/journal.pone.0256826 (PMC8396713; doi:10.1371/journal.pone.0256826)
Supplement: Attachment — Submitted filename: Response_to_Reviewers.docx [file pone.0256826.s003.docx]

**Response to reviewers**

Manuscript: Zuelke et. al., “Gender-specific design and effectiveness of non-pharmacological interventions against cognitive decline and dementia – Protocol for a systematic review and meta-analysis” (PONE-D-21-08933)

**Reviewer 1**

„I would like to thank the authors for submitting this protocol on a topic of high interest in the field. The protocol is well written, although it wasn't very clear to me what sex- or gender-specific information the authors are trying to extract from the trials, and how would these information help to improve future interventional study design? What if these information were not reported, will additional effort be made to obtain these data?”

We thank the reviewer for their kind appraisal of our manuscript! The revised manuscript includes additional information on gender-specific information to be reported in the planned systematic review. Please see page 10, lines 242ff:

“For each included study, we will provide numbers of male and female participants, gender-specific covariates and gender-specific effects of interventions. Beyond that, we will provide a narrative synthesis of gender aspects of included studies, e.g. consideration of gender in the study design, presentation of results, discussion, conclusion or recommendations, if feasible.”

Lines 264ff (changes in bold type): “Thereby, we will point out whether potential gender differences are already being taken into account in **the design and execution of** dementia prevention efforts or whether possibilities for improvement prevail. **Knowledge on whether existing intervention efforts have differential effects depending on gender is crucial for the design of future prevention strategies and personalized interventions to preserve cognitive function. Systematically assessing aspects of gender in existing trials could help facilitate the design of future interventions aimed at preserveing cognitive function. By addressing these questions, the results of our review will contribute valuable knowledge on which types of interventions work for men and women, respectively, to prevent risk of cognitive decline and dementia.”**

**Reviewer 2**

„It is a very well written project with a description of the appropriate methodology to meet the proposed objectives.”

We thank the reviewer for their kind appraisal of our manuscript!

In addition to the requested changes, we updated the information on inclusion and exclusion criteria to be applied in the planned review. In amendment to the original protocol, we will exclude studies focusing on samples with severe pre-existing conditions, e.g. myocardial infarction, cancer, coronary heart disease, history of traumatic brain injury, stroke or psychiatric diseases, e.g. major depressive disorder, as these conditions are linked to impaired cognitive function and increased risk for cognitive decline and dementia. Our systematic review and meta-analyses focusses on the prevention of age-related cognitive decline (see page 6, line 142), whereas many RCTs focusing on subjects with severe pre-existing conditions aim at rehabilitation of cognitive function. We are confident that excluding respective study samples will result in a more clearly defined samples selection.

Please see page 6, lines 139: **DELETED**: “Our review will include both RCTs investigating healthy older adults and those at-risk for dementia due to underlying medical conditions, e.g. stroke, diabetes or coronary heart disease (not limited to these conditions).”

Page 7, lines 157ff (changes in bold type): “We will exclude RCTs targeting individuals diagnosed with dementia **or focusing on samples with severe pre-existing conditions, e.g. myocardial infarction, cancer, stroke, or psychiatric diseases, e.g. major depression, as these conditions are linked to impaired cognitive function and increasedd risk for cognitive decline and dementia.”**
